# Revealing the Hemispherical Shielding Effect of SiO_2_@Ag Composite Nanospheres to Improve the Surface Enhanced Raman Scattering Performance

**DOI:** 10.3390/nano11092209

**Published:** 2021-08-27

**Authors:** Fengyan Wang, Daxue Du, Shan Liu, Linna Wang, Tifeng Jiao, Zhaopeng Xu, Haiyan Wang

**Affiliations:** 1 Hebei Key Laboratory of Applied Chemistry, Hebei Key Laboratory of Heavy Metal Deep-Remediation in Water and Resource Reuse, School of Environmental and Chemical Engineering, Yanshan University, Qinhuangdao 066004, China; fy4319@163.com (F.W.); dudx1017@163.com (D.D.); ls336300@163.com (S.L.); linnawang1@163.com (L.W.); tfjiao@ysu.edu.cn (T.J.); 2 State Key Laboratory of Metastable Material Science and Technology, School of Information Science and Engineering, Yanshan University, Qinhuangdao 066004, China

**Keywords:** SERS, LSPR, three-dimensional models, upper hemisphere shielding

## Abstract

Many studies widely used SiO_2_@Ag composite nanospheres for surface enhanced Raman scattering (*SERS*), which mainly contributes to electromagnetic enhancement. In addition to experiments, previous simulations mostly adopted a two-dimensional model in SERS research, resulting in the three-dimensional information being folded and masked. In this paper, we adopted the three-dimensional model to simulate the electric field distribution of SiO_2_@Ag composite nanospheres. It is found that when the Ag nanoparticles are distributed densely on the surface of SiO_2_ nanospheres, light cannot pass through the upper hemisphere due to the local surface plasmon resonance (LSPR) of the Ag nanoparticles, resulting in the upper hemisphere shielding effect; and if there are no Ag nanoparticles distributed densely on the surface of SiO_2_ nanospheres, the strong LSPR cannot be formed, so the incident light will be guided downward through the whispering gallery mode of the spherical structure. At the same time, we designed relevant experiments to synthesize SiO_2_@Ag composite nanosphere as SERS substrate and used Rhodamine 6G as a probe molecule to study its SERS performance. This design achieved a significant SERS effect, and is very consistent with our simulation results.

## 1. Introduction

The oxide@metal composite structures were widely investigated in various fields, such as photonic crystals, catalysis and surface enhanced Raman scattering (*SERS*), etc. [1,2,3,4] due to their outstanding physical and chemical properties [5,6,7]. The composite microspheres of oxide@metal play a significant role in SERS associated with the localized surface plasmon resonance (*LSPR*) of metal nanoparticles (*NPs*) [8,9], inducing a charge transfer effect at the oxide@metal interface [10,11]. Thus, many oxide-metal composites have been designed to improve the capabilities of SERS [12,13,14]. In recent years, SiO_2_ has attracted increasing interest due to the advantages of easy preparation and modification, high stability and low light loss [15,16,17]. Meanwhile, Ag is one of the metals with the remarkable properties of SERS because of its strong LSPR and controllable shape to generate lots of hot spots [18,19,20,21]. Therefore, in order to achieve better SERS performance, a variety of methods [22,23,24,25,26,27] were developed to prepare the SiO_2_@Ag composite nanospheres to meet the requirements of less than 10 nm among the Ag NPs gaps.

When analyzing the SERS enhancement performance of SiO_2_@Ag composite nanospheres, results are often proved by simulating the composite’s electric field distribution [22,28]. The observed high electric field intensity between Ag NPs is evenly distributed in all corners of the entire collection of SiO_2_ nanospheres. However, the structures of simulation are usually two-dimensional and the composite structure uniformly loaded with Ag NPs has significant electromagnetic enhancement everywhere. Therefore, the enhancement factors (*EF*) obtained by simulating electromagnetic enhancement are usually far from the corresponding experimental EF.

In order to improve the simulation accuracy of electromagnetic enhancement, we used the optical module of Comsol Multiphysics software to construct a three-dimensional structure of the SiO_2_@Ag composite nanosphere, and simulated the three-dimensional electromagnetic enhancement distribution of SiO_2_@Ag composite microspheres, finding that light cannot penetrate the upper hemisphere in three-dimensions due to the electrostatic shielding caused by sufficient adjacent Ag NPs, revealing SiO_2_@Ag hemisphere’s electric field electrostatic shielding effect. At the same time, we designed related experiments to prepare materials similar to the simulated structure, SiO_2_@Ag composite nanospheres were synthesized, and the effects of different methods for in-situ reduction of silver ammonia ions were characterized and analyzed. We used Rhodamine 6G (R6G) as a probe molecule to study the SERS activity of different concentrations of SiO_2_@Ag substrate, and tested its EF value to verify the simulation result. In addition, the hydrophobic properties of SiO_2_@Ag introduced in the fabricated process are studied here, which are often overlooked in SERS.

## 2. Materials and Methods

### 2.1. Materials

All chemicals were analytical grade and they were used without further purification. Tetraethyl orthosilicate (TEOS, 99.9%), ammonium hydroxide (NH_3_·H_2_O, 25–28%), silver nitrate (AgNO_3_, 99.5%), glucose (C_6_H_12_O_6_, 99.5%), and polyvinylpyrrolidone (PVP, k29–32) were purchased from Aladdin (Shanghai, China). Anhydrous ethanol (EtOH, 99.7%) was supplied from Guangfu Chemical Reagent Factory (Tianjin, China). Deionized water with a resistivity of 18.2 mΩ·cm^−1^ was obtained from a Millipore Simplicity 185 water purification system (Millipore, Co., Ltd., Bedford, MA, USA).

### 2.2. Modification of SiO_2_ Nanospheres

The monodisperse colloidal SiO_2_ nanospheres with 500 nm used in our experiments were prepared by the Stöber method [29,30]. Then, PVP was added to the aqueous solution in which SiO_2_ nanospheres were dispersed. The solution was stirred at 30 °C for 3 h. Finally, the modified SiO_2_ nanospheres were obtained through centrifugal washing with ethanol and deionized water, and vacuum drying.

### 2.3. Preparation of SiO_2_@Ag Composite Nanospheres

Firstly, the unmodified SiO_2_ or pre-modified SiO_2_ (0.1 g) nanospheres were dispersed in ethanol (13 mL). Secondly, AgNO_3_ (0.1 g) was dispersed into deionized water (2 mL) and an NH_3_·H_2_O (0.6 mL) solution to configure the silver ammonia solution. The freshly prepared silver ammonia solution was slowly added to the mixed solution with continuous stirring, and then the reducing agent was added into the mixed solution. After that, the solution was transferred into a Teflon cauldron and heated at 110 °C for 12 h. Finally, the SiO_2_@Ag composite nanospheres were obtained through centrifugal washing and vacuum drying.

### 2.4. SERS Measurement

The SiO_2_@Ag nanosphere solutions (50 µL) with the concentration of 10 mg/mL were suspended on the silicon wafer. After drying for several minutes, R6G solutions (20 µL) with concentrations ranging from 10^−10^ to 10^−6^ M were dropped on the SiO_2_@Ag substrates. The SiO_2_@Ag substrates were dried to evaporate the aqueous solution. SERS spectra were recorded using a Raman spectrometer system (Xplora plus, HORIBA, Kyoto, Japan) with a 532 nm excitation wavelength.

### 2.5. 3D Simulated Mode

**Figure 1:**
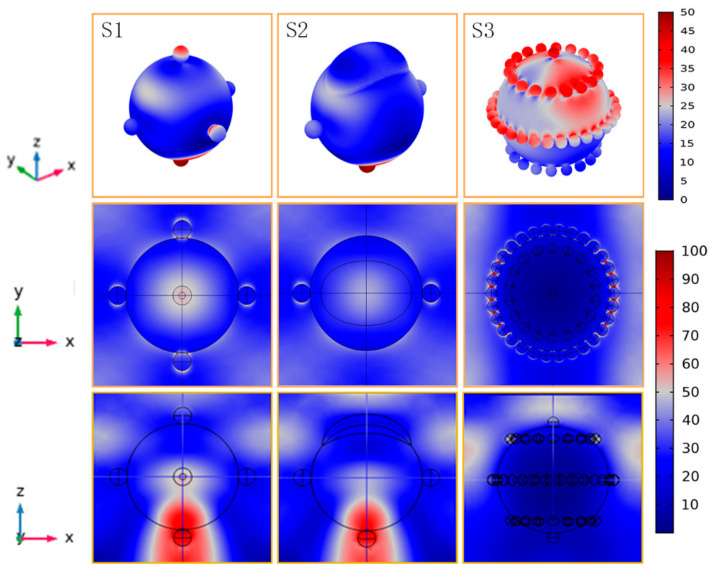
Three-dimensional electromagnetic enhancement distribution (stereoscopic view, top view and front view) of SiO_2_@Ag in S1, S2 and S3.

3D models used for simulation include material types and geometric parameters as shown in Figure 1. Since the nanospheres are hexagonally close packed, the boundary is set to periodic boundary conditions so that the minimum basic unit can accurately replace the overall situation. The minimum basic unit is set to be a core-shell structure, including a dielectric oxide nanosphere of 500 nm as core material and silver nanoparticles on its surface. The diameter of silver nanoparticles is 80 nm in S1 and S2, and it is obvious that the number of silver particles in S3 is far more than that of S1 and S2, so silver particles size is set to 60 nm. In order to simplify the calculation in S3, 1/4, 1/2, and 3/4 height regions of the core are uniformly distributed with silver nanoparticles, and the corresponding silver nanoparticles spacing is about 7.4, 18.5, and 7.4 nm. The wavelength of the incident light is 532 nm, which is consistent with the wavelength of the light source of the experimental equipment, and the generated results are the average value under TE and TM modes.

## 3. Results and Discussion

Studies have found that the SERS of SiO_2_@Ag substrate comes from electromagnetic enhancement and chemical enhancement, and the electromagnetic enhancement is absolutely dominant [31,32,33,34]. To figure out exactly how the surface electromagnetic enhancement distribution is on the SiO_2_@Ag composite nanospheres substrates, the three-dimensional SiO_2_@Ag models are constructed by Comsol Multiphysics (v. 5.3a. COMSOL AB). Ag NPs were distributed on the surfaces of a 500 nm SiO_2_ core, providing stereoscopic, top, and front views of the electric field distribution shown in Figure 1. To understand simply, S1, S2 and S3 will be respectively used to represent the three samples, where S1 represents sparse but large-sized Ag NPs, S2 is the same as S1 but with some irregular parts, and S3 is dense small-sized Ag NPs. It can be seen from the front view that the high electric field in S1 and S2 are mainly distributed directly under the SiO_2_@Ag due to the whispering gallery mode of the spherical nanostructure [15,35]. The high electric field in S3 is distributed in the gaps between the Ag NPs in the upper half of the SiO_2_@Ag mainly due to many Ag NPs with close distances confine the incident light contributed to LSPR so that most of light cannot reach the lower half of the SiO_2_@Ag.

These results are hard to spot in previous studies because the three-dimensional models reveal much of the original folded information compared to the two-dimensional models, similar to the top view of the three-dimensional models. The EF contributed by electromagnetic enhancement [34,36,37] is given by Equation (1):

(1) $$EF=\frac{I_{SERS}}{I_{RS}}\approx {\left|\frac{E}{E_{0}}\right|}^{4}$$

As shown in three views of Figure 1, the electric field intensity (*E*/*E*_0_) in S1–S3 is about 85, 78, 100 between the Ag NPs, and 2–3 times that of their surface, causing the SERS to far exceed the surrounding area. According to Equation (1), the corresponding electromagnetic enhancements are calculated to be 5.22 × 10^7^, 3.70 × 10^7^, and 1 × 10^8^, respectively.

Based on the results of the simulation, we concluded that the probe molecules need to be in contact with the bottom of the core material for a structure similar to S1 and S2, while the probe molecules need to be distributed between the Ag NPs on the upper surface for a structure similar to S3. Therefore, with SiO_2_ as the representative, we designed relevant experiments of S0, S1, S2 and S3 specifically, where S0 represents SiO_2_ nanospheres.

**Figure 2:**
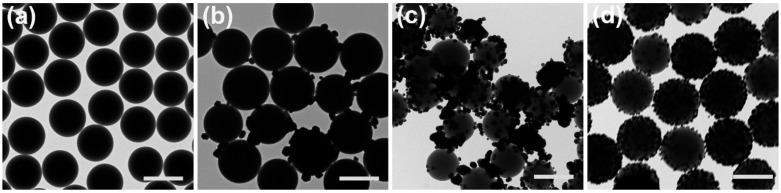
TEM images of SiO_2_ and SiO_2_@Ag nanospheres substrate with different samples: (**a**) S0, (**b**) S1, (**c**) S2, and (**d**) S3, scale bar 500 nm.

The colloidal SiO_2_@Ag composite nanospheres were first characterized by a PHILIPS TECNAI-10 transmission electron microscope (TEM), as shown in Figure 2. Figure 2a shows the pre-fabricated SiO_2_ NPs with a diameter of 500 nm used as core material to synthesize SiO_2_@Ag composite microspheres. Ag NPs supported on SiO_2_ were large and sparse, implying that a large amount of [Ag(NH_3_)_2_]^+^ ions were rapidly consumed in the solution instead of binding to the surface of SiO_2_ at 110 °C, due to the reducing ability of glucose in Figure 2b. S2 (Figure 2c) was prepared under the same conditions as S1, but using PVP pre-modifying SiO_2_ nanospheres. In comparison, S3 represents Ag NPs loading in SiO_2_@Ag obtained using PVP as the reducing agent in Figure 2d. Compared to S2, the coverage of Ag nanoparticles coated on the surface of modified SiO_2_ nanospheres dramatically improved.

**Figure 3:**
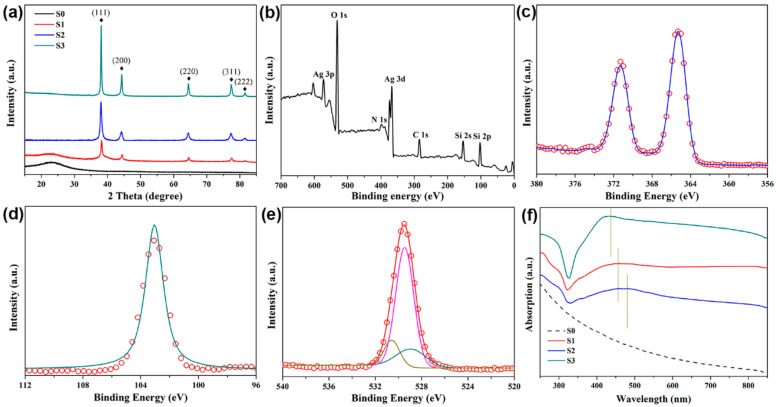
(**a**) XRD of SiO_2_ and SiO_2_@Ag composite nanospheres; (**b**–**e**) XPS analysis of SiO_2_@Ag core-shell NPs ((**b**) survey spectrum; (**c**) Ag 3d; (**d**) Si2p; (**e**) O 1s spectra); (**f**) UV-Vis absorption of SiO_2_(S0) and SiO_2_@Ag with different samples(S1–S3).

The synthesized SiO_2_@Ag nanospheres were also characterized by XRD. As exhibited in Figure 3a, the XRD patterns of the SiO_2_@Ag nanospheres displayed diffraction peaks at 2θ = 38.1°, 44.3°, 64.4°, 77.4°, 81.5° corresponding to the reflections of (111), (200), (220), (311), (222) crystalline planes of Ag (JCPDS No. 04#0783), indicating that the Ag nanoparticles were coated on the surface of the SiO_2_. Meanwhile, there appeared a broadband diffraction angle of 14.78–33.75° corresponding to the characteristic diffraction peaks of amorphous SiO_2_ (JCPDS No. 01-082-1554), and the peak intensity of SiO_2_ was suppressed after the growth of Ag. These results are in very good agreement with Figure 3.

To make clear the encapsulation state of the elements in the SiO_2_@Ag core-shell NPs, the most widely used XPS analysis was performed to elucidate the chemical composition and the interaction at the interface between Ag and SiO_2_ [6,38,39]. All binding energies were referenced to the C 1s line at 285.1 eV from carbon, and the spectrum deconvolutions were handled with the XPS Peak Fitting Program. The X-ray photoelectron spectroscopy for SiO_2_@Ag nanocomposite spheres substrates are displayed in Figure 3b–e. The Ag 3d region in Figure 3c is shown as fitted with two components, by a doublet Ag 3d_3/2_ and Ag 3d_5/2_ due to the spin-orbit coupling. The spectrum was well fitted with the typical Ag 3d doublet at the binding energies of 368.1 and 374.1 eV with the spin-orbit splitting of 6 eV. In addition, there is no other minor peak or trace of any other peaks, which reveals that the surface of Ag atoms does not oxidize and these analyses confirm the existence of metallic Ag° particles over the SiO_2_ NSs surface. We obtained a silicon dioxide signal at the binding energy of Si 2p at 103.2 eV in Figure 3d and this signal was fitted into a Gaussian curve. The fitted peak is assigned to a combination of O–Si+Si–O–Si bonds, which is in good accordance with the O 1s spectrum (Figure 3e). The deconvoluted peaks of the O 1s spectrum show the four Gaussian curves at binding energies of 530.0, 532.4, 533.5 and 534.5 eV; they are attributed to PVP, O–Si, Si–O–Si and O–C bonds, respectively. These results are in very good agreement with the XRD spectrum, with a suppression of peak intensity of amorphous SiO_2_ NPs in as obtained SiO_2_@Ag core-shell NPs. Hence, XPS analysis confirms the successful formation of SiO_2_@Ag core-shell NPs.

To understand these optoelectronic features for SiO_2_@Ag, the LSPR investigations were handled by UV–visible tests [27,40]. UV–visible absorption spectra of SiO_2_ and three kinds of SiO_2_@Ag are demonstrated in Figure 3f. The spectrum measured for bare silica powder was featureless (curve S0). However, upon the deposition of silver, the UV–visible spectrums of S1 to S3 show an adsorption peak at 451, 477 and 432 nm, due to Mie plasmon resonance excitation from the silver nanoparticles. In particular, the two red shifts, one is from 432, 451 to 477 nm; another is from 535 nm to S2’s absorption peak in the near-infrared region, and broadening width of the three main absorption peaks in UV–visible spectra were observed while the dimensions of Ag NPs were grown.

**Figure 4:**
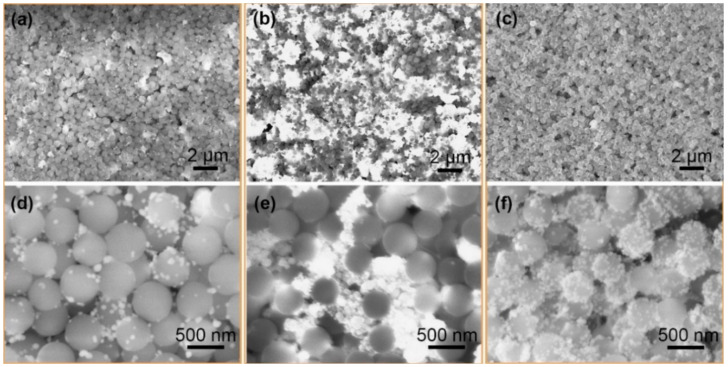
SEM images of SiO_2_@Ag nanosphere substrate with 3 different samples: (**a**) S1, (**b**) S2, and (**c**) S3; and the corresponding magnification enlarged by 6 times (**d**) S1, (**e**) S2, and (**f**) S3.

To verify the SERS activity of SiO_2_@Ag NPs, the SiO_2_@Ag was spin-coated on the silicon wafer; the associated SEM images are provided in Figure 4. Obviously, Ag nanoparticles in Figure 4a are sparse, while in Figure 4c they are uniformly scattered on the surface of SiO_2_ spheres. The difference is that in Figure 4b the Ag nanoparticles are large and sparse and some are even connected into islands. These results are consistent with TEM results, further indicating that the prepared SiO_2_@Ag nanospheres are in line with the expected simulation design.

**Figure 5:**
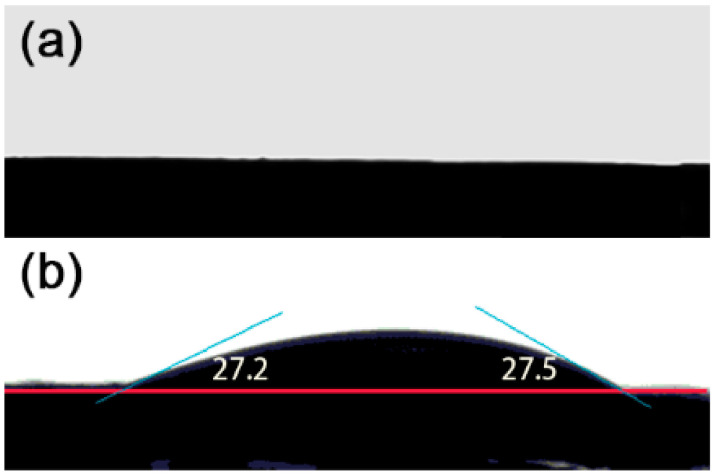
Wettability of the SiO_2_@Ag composite-nanospheres substrates: (**a**) S2, and (**b**) S3.

As shown in Figure 5, the water contact angle of S2 is basically 0, indicating that it is hydrophilic, which is the same as S1; while the water contact angle of S3 is about 27 degrees, showing a certain degree of hydrophobicity. These results suggest that R6G in the aqueous solution after evaporating solvent is more distributed on the bottom in S1 and S2, and more on the surface rather than the bottom in S3.

**Figure 6:**
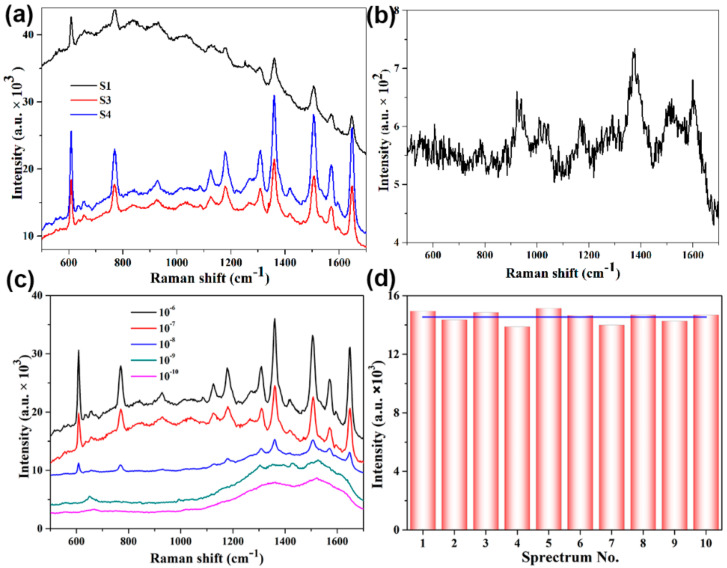
(**a**) SERS spectrum of different preparation conditions on SiO_2_@Ag substrate, (**b**) SERS spectrum of R6G with 10^2^ M adsorbed on silicon substrate, (**c**) SERS spectrum of different concentrations (10^−10^–10^−6^ M) of R6G adsorbed on SiO_2_@Ag substrate, and (**d**) Intensity distribution of R6G molecules (1 × 10^−6^ M) at the peak of 611 cm^−1^ from 10 different batches of SiO_2_@Ag nanocomposite spheres substrates.

SERS on R6G with the SiO_2_@Ag composite-nanospheres substrates were carried at 532 nm excitation wavelength. Figure 6a shows the Raman peaks of 10^−8^ M R6G spin-coated on the substrate S1, S2, S3, and Figure 6b shows the peak intensity of 0.01M R6G spin-coated on the silicon wafer. Figure 6c shows that the SERS spectra of R6G at different concentrations ranging from 10^−10^ to 10^−6^ M with the volume of 20 µL, and Raman peaks at 609, 768, 1361, 1510, 1640 cm^−1^ are clearly observed [23,41]. The spectra reveal that an R6G concentration as low as 10^−8^ M can still exhibit observable signals. The SERS enhancement factors (EF) for R6G on the SiO_2_@Ag composite-microsphere substrates can be calculated [23] according to Equation (2).

(2) $$EF=\frac{I_{SERS}/C_{SERS}}{I_{RS}/C_{RS}}$$

Correspondingly, EF was calculated within the same area of the laser spot. Meanwhile, the corresponding EF values are calculated to be about 0.81 × 10^7^, 1.43 × 10^7^, 2.41 × 10^7^ for the vibrational peak at 1361 cm^−1^. These results indicated that the synthesized SiO_2_@Ag composite nanospheres were effective and ultrasensitive when used as a SERS substrate for detection. Furthermore, the experimental results are in good agreement with the simulation results considering that the actual number and spacing of Ag NPs prepared are weaker than the ideal state in the simulation. It should be noted that there are some irregular Ag NPs of experimentally prepared S2, hot spots between such Ag NPs causing electromagnetic enhancement, but these structures are simplified into a smooth block without these hot spots in the simulation model. Therefore, it causes a slight mismatch between the experimental and simulated values.

The good conformity and reproducibility of the SERS signal are also essential for as-prepared SiO_2_@Ag SERS substrates. In this work, the SERS enhancement distributed on the surface was measured by randomly collecting the SERS signal intensity from 10 different batches of SiO_2_@Ag nanocomposite spheres substrates. Figure 6d shows the intensity distribution of the peaks at 611 cm^−1^ of the R6G with a concentration of 10^−6^ M collected on 10 different batches of SiO_2_@Ag substrates; the blue line shows the average intensities. The relative standard deviation (*RSD*) value of the peaks at 611 cm^−1^ is 0.03, indicating that intensities of the SERS spectra with little fluctuations around the average intensity are almost similar. The deviation required by actual SERS measurement was less than 0.2 [42], and the SiO_2_@Ag base prepared in this paper could meet the needs of practical applications. Therefore, the SiO_2_@Ag SERS substrates with ultrahigh activity and reproducibility have potential ability in practical applications.

By comparing and analyzing the calculated results with the experimental results, it is found that due to the shielding effect of the upper hemisphere, the reagents in the lower hemisphere were ineffective. Therefore, if the hydrophobic surface could be designed during the experiment, not only could the amounts of reagents be saved, but the SERS performance could also be improved.

## 4. Conclusions

In summary, this work simulated the three-dimensional electromagnetic enhancement distribution of SiO_2_@Ag composite microspheres, revealing SiO_2_@Ag hemisphere’s electric field electrostatic shielding effect. The simulation results illustrated that the surface electromagnetic enhancement distribution of SiO_2_@Ag nanocomposite spheres could be tuned by altering the number of Ag NPs coated on the surface of the dielectric Oxide nanospheres. Additionally, the upper hemispherical shielding effect is caused by the LSPR of a large number of Ag NPs, which does not depend on the core materials. In order to verify the calculation results, SiO_2_@Ag composite nanospheres were synthesized and the SERS performance of SiO_2_@Ag composite nanospheres substrate was studied. The experimental results are in good agreement with the simulated ones, although there is a slight mismatch between the experimental and simulated values in S2; this is because there are some irregular Ag NPs in the experiment, and the hot spots between these Ag NPs lead to electromagnetic enhancement, but in the simulation model these structures are simplified to a light slider, without these hot spots. When the Ag NPs distribution state is S3, the SiO_2_@Ag nanocomposite-spheres substrate has the best SERS performance, with an EF value of 2.41 × 10^7^. By comparing and analyzing the calculated results with the experimental results, it is found that the obtained SiO_2_@Ag nanocomposite spheres substrates have high sensitivity, uniformity, reproducibility and therefore they have huge potentials for qualitative and quantitative analytical detection. However, due to the hemispherical shielding effect, the probe molecules falling below the SiO_2_@Ag lose their effect and cause weak Raman signals. In order to further improve SERS performance and save reagent consumption, the SERS base can be designed as a hydrophobic surface, and this model can be extended to other Oxide@Ag nanocomposite spheres substrates.

## Data Availability

The data is not available due to further study.
